# Technological Innovations for Improving Cassava Production in Sub-Saharan Africa

**DOI:** 10.3389/fgene.2020.623736

**Published:** 2021-01-21

**Authors:** Edwige Gaby Nkouaya Mbanjo, Ismail Yusuf Rabbi, Morag Elizabeth Ferguson, Siraj Ismail Kayondo, Ng Hwa Eng, Leena Tripathi, Peter Kulakow, Chiedozie Egesi

**Affiliations:** ^1^International Institute of Tropical Agriculture, Ibadan, Nigeria; ^2^International Institute of Tropical Agriculture, Nairobi, Kenya; ^3^CGIAR Excellence in Breeding Platform, El Batan, Mexico; ^4^National Root Crops Research Institute, Umudike, Nigeria; ^5^Department of Global Development, College of Agriculture and Life Sciences, Cornell University, Ithaca, NY, United States

**Keywords:** cassava, innovations, QTL mapping, association mapping, marker-assisted selection, genomic selection, genome-editing

## Abstract

Cassava is crucial for food security of millions of people in sub-Saharan Africa. The crop has great potential to contribute to African development and is increasing its income-earning potential for small-scale farmers and related value chains on the continent. Therefore, it is critical to increase cassava production, as well as its quality attributes. Technological innovations offer great potential to drive this envisioned change. This paper highlights genomic tools and resources available in cassava. The paper also provides a glimpse of how these resources have been used to screen and understand the pattern of cassava genetic diversity on the continent. Here, we reviewed the approaches currently used for phenotyping cassava traits, highlighting the methodologies used to link genotypic and phenotypic information, dissect the genetics architecture of key cassava traits, and identify quantitative trait loci/markers significantly associated with those traits. Additionally, we examined how knowledge acquired is utilized to contribute to crop improvement. We explored major approaches applied in the field of molecular breeding for cassava, their promises, and limitations. We also examined the role of national agricultural research systems as key partners for sustainable cassava production.

## Introduction

The agricultural sector is key to economic growth in Africa. The recent report on the Global Hunger Index indicates that over half of the world’s food-insecure people live in Africa (FSIN, 2019). Sustainable agricultural production is imperative to curb food insecurity, reduce poverty, and impact the livelihood of smallholder farmers (Ojijo et al., 2016; Donkor et al., 2017). Cassava is among the six commodities defined by the African Heads of States as strategic crops for the continent, given its significant contribution to the livelihoods of African farmers and its potential for transforming African economies (Feleke et al., 2016).

Cassava (*Manihot esculenta* Crantz) is a root crop grown throughout the tropics by more than 800 million people (Nassar and Ortiz, 2010). It can grow with minimal inputs under marginal soil conditions and in regions prone to drought. Though mainly cultivated for its starchy roots, nutrient-dense cassava leaves are also consumed as vegetables in many regions of Africa (Spencer and Ezedinma, 2017). Due to its long harvest window, cassava roots are used as a food reserve during periods of food shortage or during the lean season before harvest of other crops. Although its cultivation has traditionally been associated with subsistence farming, the crop is gradually becoming an industrial crop, which is processed into different products, including bread, pasta, and couscous-like products (Bechoff et al., 2018; Mtunguja et al., 2019). Apart from the food industry, cassava starch is used for textiles, the paper industry, in the manufacture of plywood and veneer adhesives, glucose and dextrin syrups (Tonukari et al., 2015; Spencer and Ezedinma, 2017; Waisundara, 2018).

Sub-Saharan Africa accounts for 61.1% of the world’s cassava production (FAOSTAT, 2020). Although some increase have been achieved recently, mainly due to expansion in crop area, cassava average productivity (9.1 t/ha) on the continent is well below the average cassava fresh root yield (21.5 t/ha) recorded in Asia (Spencer and Ezedinma, 2017; FAOSTAT, 2020). In order to satisfy the forecasted increase in demand for cassava food and non-food products, and to harness the enormous potential offered by the crop, cassava production in sub-Saharan Africa must be increased (Khandare and Choomsook, 2019; Otekunrin and Sawicka, 2019; FAOSTAT, 2020). Some of the constraints inherent to its production include pests (Kalyebi et al., 2018; Koros et al., 2018) and diseases caused by bacteria (Fanou et al., 2018) and viruses (Patil et al., 2015; Alicai et al., 2019) leading to significant yield losses. Another drawback is the cyanogenic glucosides, which upon hydrolysis, produces toxic hydrogen cyanide (Akinpelu et al., 2011). Cassava is an energy-dense food, mainly composed of starch with low levels of protein and other nutrients important for a balanced diet. Populations that consume cassava as a staple are at high risk of protein, vitamin A, zinc, and/or iron deficiency (Gegios et al., 2010; Stephenson et al., 2010). These challenges guide the breeding objectives: (a) high yield in terms of dry matter per unit land area; (b) resistance to diseases, such as cassava mosaic disease (CMD), cassava brown streak disease (CBSD), and cassava bacteria blight (CBB), and pests such as cassava green mites (CGM) and whiteflies; (c) improved starch quality and quantity; (d) low hydrogen cyanide potential; (e) improved nutritional value or biofortification; (f) adaption to a wide range of environments; (g) improved plant type for mechanization; and (h) end user characteristics, including processing, cooking, and organoleptic properties (Teeken et al., 2020).

Conventional breeding has been efficient in providing a continuous supply of improved cultivars that have resulted in a dramatic increase in yield of most major crops (Prohens, 2011). Conventional cassava breeding is based on phenotype-based recurrent selection, which relies on the production of full-sib and/or half-sib progenies followed by successive clonal selection stages, including single row trials, preliminary, advanced, and uniform yield trials (Ceballos et al., 2016). Many cassava varieties have been developed and released through conventional breeding (Malik et al., 2020). Breeding cassava is a challenging task due to the heterozygous genetic make-up of the crop. The development of improved varieties is time consuming due to its long breeding cycle (12 months). New tools and technologies have the potential to improve the efficiency of conventional breeding, especially when several traits are being selected at the same time. Modernization of breeding programs, through the application of innovative tools, is vital for more efficient agriculture, especially in the context of climate change, shrinking resources, land scarcity, and increased food demand. Biotechnology and new genomic approaches have the potential to enhance genetic gain, speed up the development of better cultivars, and impact the livelihoods of smallholder farmers.

We highlighted the genomic resources available in cassava and their potential applications. We also reviewed the state of knowledge of the phenotyping technologies currently available and their shortcomings. Additionally, we explored the potential and implications for integration of technological innovations in cassava genetics and breeding. With farmers being the ultimate beneficiaries, we examined how to ensure regular, sustainable high level of cassava production in sub-Saharan Africa; thereby, contributing to food security challenges and improved livelihoods through income generation.

## Genomic Resources and Their Use

Genomic resources for cassava have increased substantially in recent years. Thousands of simple sequence repeats (SSR) markers have been developed from expressed sequence tags and enriched genomic DNA libraries (reviewed in Ferguson et al., 2011). The cassava chromosome-scale reference genome and the re-sequencing of diverse accessions, has identified a large number of sequence polymorphisms. Single nucleotide polymorphisms (SNPs), methylation polymorphism, Insertions-Deletions (Indels), and structural variants have been identified (Sakurai et al., 2013; Wang et al., 2014; Xia et al., 2014; Bredeson et al., 2016). A cassava haplotype map harboring 25.9 million SNPs and 19 million indels has been developed (Ramu et al., 2017). High-throughput genotyping platforms (e.g., GoldenGate assay) have allowed researchers to simultaneously interrogate tens of thousands of SNPs at a reduced cost (Ferguson et al., 2012). The development of low cost genotyping technologies based on multiplex sequencing platforms, such as genotyping by sequencing, has enabled rapid and accurate high-density fingerprinting using SNP markers (Elshire et al., 2011; ICGMC, 2015; Rabbi et al., 2015; Kamanda et al., 2020)5). These resources provide valuable tools that have contributed to genetic research and molecular breeding of cassava.

### Genetic Diversity

Genetic diversity is of paramount importance in crop improvement through breeding. The extent and nature of genetic variation existing within cassava landraces and cultivars from selected African countries have been assessed using molecular markers (Kawuki et al., 2009; Kamanda et al., 2020). These studies have revealed genetic differentiation between African and South American germplasm, as well as differentiation between cassava landraces within Africa. Those from East, South, and Central Africa are somewhat differentiated from those from West Africa (Kawuki et al., 2009; Ferguson et al., 2019; Adjebeng-Danquah et al., 2020). The narrow genetic base of African cassava breeding lines is attributable to intense selection pressure for CMD resistance with recurrent selection using few parents and the clonal nature of the crop (Turyagyenda et al., 2012; Wolfe et al., 2016; Ferguson et al., 2019). Slightly higher genetic diversity has been reported in landraces in comparison to elite accessions (Ferguson et al., 2019), which is typical of most crops. It is likely that the genepool of pre-breeding germplasm will become more diverse as breeders begin to incorporate variation that responds better to consumer preferences.

### Genetic Redundancy and Breeding Impact

Fingerprinting of cassava accessions using molecular markers has many applications. Genetic redundancy represents a challenge to efficiently manage and optimize the conservation of genetic resources in genebanks and breeders’ collections. SNP markers have been used to confirm that particular accessions are not identical, and others are possible duplicates (Ferguson et al., 2012). They have also been used to assess the adoption of improved varieties (Turyagyenda et al., 2012; Rabbi et al., 2015; Wossen et al., 2017). Molecular markers have an important role to play as farmers frequently give different names to the same cultivar or landrace. Thus, they become difficult to identify, particularly as cassava varieties are not easy to distinguish morphologically. This enables the correct assessment of adoption rates, which in turn, influences breeding priorities and agricultural policies (Kretzschmar et al., 2018). Molecular markers have also been used to assess the integrity of putative mapping populations, select true-cross progeny, validate crosses, and guide parental selection (Rabbi et al., 2012; ICGMC, 2015; Masumba et al., 2017).

## Phenotyping of Key Traits

Phenotyping is important to support crop breeding. The acquisition of phenotype data remains a bottleneck hindering cassava genetic studies and full-deployment of genomics-assisted breeding. Several approaches have been used to phenotype breeding lines and germplasm collections for nutrition (carotenoids, cyanogenic potential), yield and yield components (dry matter content), quality (starch physiochemical and functional properties, texture, and pasting properties), biotic stresses (disease resistance), and root system architecture. In the following section, we detail the current phenotyping strategies used in cassava.

### Carotenoids

Breeding cassava roots with enhanced levels of provitamin A carotenoids is a high priority in some breeding programs. The color of the cassava storage root parenchyma has been correlated with the total carotenoids content; thus, used to evaluate carotenoids content (Iglesias et al., 1997; Afonso et al., 2017). A challenge has been to efficiently distinguish the subtle differences within each color group, as this is difficult by eye. To overcome this limitation, quantification of total carotenoids content by ultraviolet-visible spectrophotometry, as well as identification and quantification of β-carotene and its isomers by high-performance liquid chromatography (HPLC), have been employed (Carvalho et al., 2012; Belalcazar et al., 2016). These approaches are accurate, but have many drawbacks, including cost, time-needed for analysis, labor-intensive methods, and requirements for laboratory infrastructure and trained technical staff, which is not always available to breeding programs in Africa (Udoh et al., 2017). Alternative portable devices, such as ICheck^TM^ carotene, have been proven useful for rapid field evaluation and could be valuable in remote areas with no laboratory facilities or electricity (Esuma et al., 2016; Jaramillo et al., 2018). Color instruments designed to quantify the *Commission International de l’Eclairage* (CIELAB) color parameters have also been successfully used to evaluate carotenoids content in cassava root samples in an efficient way (Afonso et al., 2017). Near-infrared spectroscopy (NIRS) is another promising approach that has been explored for carotenoids quantification with demonstrated high prediction accuracy (Sánchez et al., 2014; Ikeogu et al., 2019).

### Cyanogenic Potential

Cassava contains naturally occurring, but potentially toxic compounds called cyanogenic glycosides, which release hydrogen cyanide (HCN) on hydrolysis. This can be highly toxic to humans and animals if not removed through processing. It is important that the levels of cyanogenic glycosides are measured. Several approaches have been used to quantify cyanide potential, including the titration method (Moriasi et al., 2017; Iliya and Madumelu, 2019), the alkaline picrate method (Fukushima et al., 2016; Moriasi et al., 2017), and the metal-based chemosensors (Tivana et al., 2014). These approaches involved multi-step reactions and necessitate trained personnel. The picrate method, although easy to use, is very slow (minimum 12 h) and the chemicals used are hazardous. Recently, it was shown that NIRS could efficiently be used to distinguish roots with high or low cyanogenic potential (Sánchez et al., 2014).

### Dry Matter Content

Storage root dry matter content (DMC) reflects the proportion of useable fresh root yield. DMC is commonly measured using either specific gravity through suspension of a root sample in water and air, or the oven-drying method, which has been the most widely used method. This is where a representative root sample is weighted wet and then oven dried to constant weight (Fukuda et al., 2010; Teye et al., 2011). The oven-drying method is tedious when working with a large number of samples. Likewise, it is difficult to implement this approach where the source of electricity is unreliable (Teye et al., 2011). Both oven-drying and specific gravity could be substituted by NIRS, which has been shown to predict DMC with a high degree of accuracy (Belalcazar et al., 2016).

### Starch Physiochemical and Functional Properties

Physiochemical properties among cassava cultivars determine root quality attributes important for processing and consumption. The main constituent of cassava storage roots is starch, which is composed of amylose and amylopectin. Both of these play a crucial role in retrogradation, gelling, pasting, crystallinity, gelatinization temperature, viscosity, texture, cooking, eating, and processing quality of cassava (Ayetigbo et al., 2018). Amylose content (AC) is the most important factor influencing cooking and textural quality. The AC in cassava has been estimated using iodine colorimetry (Sandoval-Aldana and Fernandez, 2013; Boonpo and Kungwankunakorn, 2017) or Megazyme amylose/amylopectin assay kit (Chisenga et al., 2019). Iodine colorimetry is prone to inter-laboratory variability due to the complexity of the procedure and relies on the development of a suitable curve of known amylose to-amylopectin ratios. The swelling power of starch determines its specific functional properties when utilized in food products (Noranizan et al., 2010). Swelling power and solubility patterns of cassava flour have been determined using the Leach (1959) and Kainuma et al. (1967) method, respectively (Chisenga et al., 2019; Ma’Aruf and Abdul, 2020). Starch gelatinization properties (onset, peak and conclusion gelatinization temperature, and enthalpy) and retrogradation have been determined using differential scanning calorimetry (DSC). DSC can be run at a rate of four samples per hour (Thirathumthavorn and Trisuth, 2008; Tappiban et al., 2020). Crystallinity measurement requires the use of an x-ray diffractometer, a piece of complex and expensive equipment (Chatpapamon et al., 2019). In sweet potato, it was shown that NIRS could predict most physiochemical and thermal properties of starch with acceptable precision (Lu et al., 2006a,b). NIRS was sufficiently accurate for the determination of total starch and amylose in barley in the study of Ping et al. (2013). Meanwhile, Cozzolino et al. (2013) demonstrated that swelling properties and water solubility could be determined in whole grain barley using NIRS spectroscopy. NIRS technology could potentially be used to predict functional and physiochemical properties of cassava or cassava-based products.

### Texture and Pasting Properties

Texture is a critical factor for consumer acceptance of cassava. Sensory analysis has been used for characterizing cassava and cassava-based product texture properties. The sensory descriptors assessed included texture, appearance, odor, taste, masticability (Akely et al., 2016; Adinsi et al., 2019). The cost associated with training and maintaining a descriptive panel combined with the low throughput of sensory evaluations has prompted the development of less costly and less time-consuming approaches. Instrumental methods using a texture analyzer that mimics mastication have been used to evaluate and/or predict the texture of raw, cooked, and processed cassava products. The parameters measured include hardness, springiness, adhesiveness, gel strength, mouldability, elasticity, smoothness, appearance, thickness, and general acceptability (Rodríguez-Sandoval et al., 2008; Maieves et al., 2012; Rosales-soto et al., 2016; An et al., 2019; Ma’Aruf and Abdul, 2020). Pasting properties of cassava products are key determinants of quality. Rapid Visco Analyzer (RVA) has been used to evaluate pasting properties of cassava accessions, and the parameters estimated include peak viscosity, setback viscosity, final viscosity, pasting temperature, and time to reach peak viscosity. Using RVA, less than five samples can be processed in 1 h (Rosales-soto et al., 2016; Chatpapamon et al., 2019). Although not yet reported for cassava, NIRS has been shown to adequately predict texture and pasting properties of rice (Meullenet et al., 2002; Chueamchaitrakun et al., 2011) and sweet potato (Lu et al., 2006a,b). Therefore, the ability of NIRS to predict cassava pasting properties should be explored.

### Biotic Constraints

Different purposes required different discrimination methods for quantification of disease incidence and/or severity. For example, for breeding purposes, a visual scale of 1–5 from disease-free to highly diseased may be sufficient, where breeders will only retain those cultivars with a score lower than two. However, this is subjective and dependent upon personal perceptions. On the other hand, QTL mapping or other applications may require greater resolution or more accuracy (Garcia-Oliveira et al., 2020). Accurate evaluation and novel approaches for cassava disease detection are needed to efficiently assess disease severity (Chiang et al., 2016). Root necrosis indexes for CBSD evaluation, which account for the root sample size or their economic value, have been proposed to replace traditional based evaluation (Kawuki et al., 2019). Image analysis has similarly been used (Garcia-Oliveira et al., 2020; Nakatumba-Nabende et al., 2020). Analysis of field images combined with various algorithms, including K mean clustering algorithms (Anderson et al., 2015), artificial neutral network (Abdullakasim et al., 2011), and more recently, machine learning techniques and convolutional neutral network (Owomugisha and Mwebaze, 2016; Sambasivam and Opiyo, 2020) have provided a more accurate and objective assessment of disease severity and incidence. The smart phone-based diagnostic system (NURU-AI) is being developed to support remote diagnosis by smallholder farmers in Africa for real-time prediction of the state of cassava health (Owomugisha and Mwebaze, 2016; Ramcharan et al., 2017, 2019).

### Root System Architecture

Uniformity in size and shape of cassava roots is an important breeding objective. Routine assessment of storage root size and shape have relied on visual scores that are both subjective, time consuming, labor-intensive and destructive. Implementation of non-invasive approaches include image analysis of roots to provide unbiased quantitative data on important root traits. High throughput three-dimensional imaging and non-destructive methods such as ground penetrating radar (GPR) have recently been developed and used for quantifying cassava storage roots and their parameters for estimation of size and shape under field condition (Delgado et al., 2017, 2019). Kengkanna et al. (2019) developed cassava shovelomics to evaluate root architecture traits. More recently, Yonis et al. (2020), demonstrated the feasibility of root phenotyping using image capture and analysis. Routine implementation of these new phenotyping solutions offers new opportunities for cassava breeders to efficiently and precisely select and release cultivars with root architecture that are favorable for harvesting, processing, as well as selection for early bulking characteristics. The development of robust phenotyping technologies for large-scale phenotyping with increased precision at reduced cost will enable efficient screening of larger populations. Precise phenotyping is invaluable for carrying out downstream analysis, including characterization of genetic factors that contribute to phenotypic variation.

## Connecting Genotypic and Phenotypic Information and Discovery of Useful Genes and/or Quantitative Trait Loci

Genotype variations have been linked to corresponding differences in phenotypes and the genetic basis of the phenotypic variability evaluated to identify genes and/or quantitative trait loci (QTLs) associated with the traits of interest. Two approaches have been used for genotype-phenotype association: (1) classical QTL mapping using experimental populations derived from bi-parental crosses with contrasting phenotypes (Collard et al., 2005); and (2) genome-wide association studies (GWAS) mapping that use germplasm collections and incorporate historical events that have occurred during domestication of the crop. Classical QTL analysis in cassava has been made possible with the development of numerous genetic linkage maps (Table 1) and use of statistical approaches that have resulted in several QTLs identified for economically important traits (Table 2). With the decreasing cost of DNA sequencing, new genotyping technologies and their improved accuracy, GWAS is being increasingly used for genetic analysis of traits. GWAS has enabled the exploration of allelic diversity that exists in natural populations, the discovery of beneficial alleles in several crops. Marker-trait associations in cassava have focused on a few important traits of interest.

**TABLE 1:** Summary of the published genetic linkage maps of cassava.

Population	Mapped markers	Linkage groups/chromosomes	Map length (cM)	References
TMS30572 (♀) × CM2177-2 (♂) F_1_ (*n* = 90)	32 RFLPs, 30 RAPDs, 3SSRs, 3 Isoenzymes	20	932.6 (female)	Fregene et al., 1997
	107 RFLPs, 50 RAPDs, 1 SSR	24	1220 (male)	
TMS30572 (♀) × CM2177-2 (♂) F_2_ (*n* = 268)	100 SRRs	22	1236.7	Okogbenin et al., 2006
Huay Bong × Hanatee F_1_ (*n* = 100)	510 SSRs	23	1420.3	Sraphet et al., 2011
Hanate (♀) × Huay Bong (♂) F_1_ (*n* = 100)	303 SSRs	27	1328	Whankaew et al., 2011
Namikonga (♀) × Albert (♂) F_1_ (*n* = 130)	434 SNPs, 134 SSRs	19	1837 (one step)	Rabbi et al., 2012
	348 SNPs, 128 SSRs	18	1541 (two steps map)	
IITA-TMS-4(2)1425 (♂) x IITA-TMS-011412 (♂) F_1_ (*n* = 180)	6756 SNPs	19	3771	Rabbi et al., 2014
KU50 × SC 124 F_1_ (*n* = 85)	2331 SNPs, 537 Indels, 164 methylation markers	20	1970.40	Xia et al., 2014
Huay Bong (♀) × Hanatee (♂) F_1_ (*n* = 100)	2110 SNPs	19	1785.62	Pootakham et al., 2014
TMS30572 (♀) × CM2177-2 (♂) F_1_ (*n* = 132)	2141 SNPs	18	2571	Soto et al., 2015
9 bi-parental F_1_ crosses 1 self-pollinated cross (S1) *n* = 1740	22,403 SNPs	18	2412	ICGMC, 2015
Kibora (♀) × AR37-80 (♂) F_1_ (*n* = 106)	1974 SNPs	21	1698	Nzuki et al., 2017
Namikonga (♀) × Albert (♂) F_1_ (*n* = 240)	943 SNPs	18	1776.2	Masumba et al., 2017
AR40-6 (♀) × Albert (♂) F_1_ (*n* = 130)	2125 SNPs	18	1730	Garcia-Oliveira et al., 2020

*SNP, single nucleotide polymorphism; RAPD, random amplified polymorphism DNA; RFLP, restriction fragment length polymorphisms; SSR, simple sequence repeat.*

**TABLE 2:** Summary of the QTL studies of cassava using controlled populations.

Traits	Mapping Population	Markers Used	Key Findings	References
CBB	TMS 30572 (♀) × CM 2177-2. (♂) F_1_ (*n* = 150)	68 RFLPs (female map) 15 RFLPs (male map)	Eight (8) QTLs identified on five LGs in the female map explaining 9 - 20%. In the male map, four QTLs explaining 10.7–27.1% of the PVE.	Jorge et al., 2000
CBB	TMS 30572 (♀) × CM 2177-2 (♂) F_1_ (*n* = 150)	Female map (95 RFLPs, 36 RAPD, 3 isoenzymes, 3 SSRs, 3 EST, 2 unknown genes) Male map (89 RFLPs, 41 RAPDs, 4 unknown genes, 1 EST)	Eight (8) QTLs detected. Alleles coming from both parents contribute to the resistance in the progenies.	Jorge et al., 2001
CMD	TME 3 × TMS 30555 F_1_ (*n* = 80)	186 SSRs	First report of the *CMD2* locus associated with CMD resistance and identification of SSRs linked to the CMD resistance genes *CMD2.*	Akano et al., 2002
DMC	MCOL 1684 × THAI 1 F_2_ (*n* = 199)	98 SSRs	Six (6) QTLs on four different linkage groups explaining 14 to 40% of the PVE.	Kizito et al., 2007
Cyanogenic glycoside (CN)			Two (2) QTLs on two different LGs explaining 7% and 20% of the PVE, respectively.	
Anthracnose	TME 117 (♀) × TMS 92/0326 (♂) F_1_ (*n* = 60)	53 RAPDs	Using bulk segregant analysis, two markers (OPAF2 and OPF06) flanking the *CAD1* genes were identified that explained 33.7 and 27.4% of the phenotypic variation.	Akinbo et al., 2007
Fresh foliage	TMS 30572 (♀) × CM 2177-2 (♂) F_2_ (*n* = 268)	122 SSRs	Three (3) QTLs for fresh foliar detected on three linkage groups. The PVE ranged from 5.5 to 31.1%.	Okogbenin et al., 2008
Harvest index			Three (3) QTLs associated with harvest index on three LGs explaining 2.10–6.67% of the phenotypic variation. QTL with major effect accounted for 36.9%.	
Dry yield root			Three (3) QTLs for dry root yield on three LGs (1, 3, 13) accounting for 6.0–9.2% of the PVE.	
Cyanogenic potential	Hanatee (♀) × Huay Bong (♂) F_1_ (*n* = 100)	303 SSRs	Five (5) QTLs mapped on LG2, 5, 10, 11 explained 16.1 to 26% of the PVE. The most significant association was on LG10.	Whankaew et al., 2011
CGM	Four backcross populations (CW 65, 66, 67, 68)	500SSRs	Using bulk segregant analysis, three markers (SSRY11, SSRY 346, and NS1099) associated with CGM resistance were detected.	Choperena et al., 2012
Starch pasting viscosity	Hanatee (♀) × Huay Bong (♂) F_1_ (*n* = 100)	510 SSRs	Fifteen (15) QTLs affecting five starch pasting viscosities identified when average values of the three environments were used. The PVE ranged from 10.0 to 48.4%. Data from separate environment revealed 48 QTLs for seven starch pasting properties, explaining 6.6 to 43.7% of the phenotypic variation.	Thanyasiriwat et al., 2013
Starch pasting properties	Huag Bong (♀) × Hanate (♂) F_1_ (*n* = 100)	2110 SNPs	Single co-localize QTL for pasting temperature and pasting time detected on LG7. The PVE explained was 44.7 and 24.3% for pasting temperature and pasting time, respectively. Additional QTL for starch pasting time found on LG10 (PVE = 22.5%).	Pootakham et al., 2014
CMD	IITA = TMS-4(2) 1425 (♂) x IITA-TMS-011412 (♂) F_1_ (*n* = 108)	6756 SNPs	Single SNP underlying CMD resistance in the vicinity of the previously mapped *CMD2* locus that explain 74% of the variance	Rabbi et al., 2014
CMD	TMS961089A × TMEB117	4394 SNPs	Single SNP underlying CMD resistance in the vicinity of the previously mapped *CMD2* locus that explain 74% of the variance	Echefu et al., 2016
Fresh starch content	Huag Bong (♀) × Hanate (♂) F_1_ (*n* = 100)	510 SSRs	Nine (9) QTLs on seven LGs, including 6, 7, 9, 11, 13, and 11 that explained 11.3 to 27.3% of the phenotypic variation. Six of the QTLs were location specific while two were found across environments.	Sraphet et al., 2017
CBSD root necrosis	Namikonga (♀) × Albert (♂) F_1_ (*n* = 240)	943 SNPs	Two QTLs consistent across seasons/sites on Chr11 and 2 and a putative QTL on Chr18. Further additional QTLs on Chr3, 4, 5, 6, 7, 10, 12, 15, and 16	Masumba et al., 2017
CBSD foliar symptoms			Several QTLs revealed on all chromosomes. The most interesting QTL was on Chr2 and has a PVE of 4.6%.	
CMD			Two (2) QTLs identified on Chr12 that explain 16.43 and 13.5% of the phenotypic variation. Additional QTLs detected on all other chromosomes except Chr11 and 14.	
CBB	TMS30572 × CM2177-2 F_1_ (*n* = 117)	2571 SNPs	Five-strain specific QTLs for resistance to *Xam* explaining between 15.8 and 22.1% of the PVE	Sedano J. C. S. et al., 2017
CBSD root necrosis	Kibora (♀) × AR37-80 (♂) F_1_ (*n* = 106)	1974 SNPs	Three (3) QTL associated with CBSD root necrosis on Chr5, 11, and 12 explaining 2.62–10.18% of the phenotypic variation	Nzuki et al., 2017
CBSD foliar symptoms			A total of seven QTLs detected on Chr4, 6, 17 and 18 explaining 2.64–8.45% of the PVE	
CMD			Two (2) QTLs detected on Chr12 and Chr14 with a maximum PVE of 13.01 and 13.36%, respectively.	
Leaf pubescence	TMEB778 (♀) × TMEB419 (♂) F_1_ (*n* = 109)	42204 SNPs	Seventy-one (71) SNPs significantly associated with leaf pubescence on Chr12. The top significant SNP explained 26% of the phenotypic variation.	Ezenwaka et al., 2020
Stay green			A total of 126 SNPs associated with stay green. The PVE ranged from 20 to 30%.	
CMD	AR40-6 (♀) × Albert (♂) F_1_ (*n* = 130)	2125 SNPs	Provide evidence for two QTL or multi-allelic variants influencing CMD resistance within the locality of *CMD2* locus. Additional putative QTLs on Chr 9 and 10	Garcia-Oliveira et al., 2020
CGM			Five (5) minor effect QTLs specific to the planting stage. Four QTLs detected at 3 MAP on Chr 5, 9, 13, and 18. PVE ranging from 1.35 to 11.06%, while one QTL detected on Chr16 at 6 MAP accounting for 7.27% of the PVE.	
CBSD foliar symptoms			One minor QTL on Chr11 accounting for 2.5 % phenotypic variation. One major QTL identifying on Chr18 explaining 12.87% of the phenotypic variation	
CBSD root necrosis			New putative QTL for CBSD root necrosis identified on Chr14	

*SNP, single nucleotide polymorphism; RAPD, random amplified polymorphism DNA; RFLP, restriction fragment length polymorphisms; SSR, simple sequence repeat; EST, expressed sequence tag; CBSD, cassava brown streak disease; PVE, phenotypic variance; LG, linkage group; Chr, Chromosome.*

### Cassava Mosaic Disease

The dominant gene *CMD2* underlying CMD resistance was discovered in the farmer-preferred Nigerian landrace TME 3 (Akano et al., 2002). A new linked QTL underlying CMD resistance named *CMD3* that explained 11% of the phenotypic variance (PVE) was later identified 36 cM away of *CMD2* gene by Okogbenin et al. (2012). The qualitative nature of CMD resistance was confirmed in subsequent studies, and a single locus with a large effect in the vicinity of the previously mapped *CMD2* locus was uncovered (Rabbi et al., 2014; Echefu et al., 2016; Table 2). The first GWAS mapping study in cassava conducted by Wolfe et al. (2016) identified 198 significant SNPs associated with CMD severity on 14 chromosomes. The significant SNPs were mostly concentrated in a single region on chromosome 8 (based on *Manihot esculenta* v5 genome assembly corresponding to chromosome 12 on v5.1 and v6.1) that account for 30 to 60% of variation in genetic resistance. Additional regions with small effects, including one on chromosome 9 that co-located with the *CMD1* resistance were also reported (Wolfe et al., 2016; Table 3). The study of Wolfe et al. (2016) substantiated bi-parental mapping studies (Akano et al., 2002; Okogbenin et al., 2012) reporting the single major gene, *CMD2*, determining CMD resistance and a second QTL, *CMD3*, closely linked to *CMD2*. A key outcome of the Wolfe et al. (2016) study was the lack of other major-effect loci. Likewise, significant interactions between the significant SNPs were disclosed. Nzuki et al. (2017) found two QTLs associated with CMD on chromosomes 12 and 14. The percentage of variation explained (PVE) by these QTLs were 13.01 and 13.36%, respectively. The QTL on chromosome 12 was confirmed as the QTL linked to the *CMD2* locus. Masumba et al. (2017) detected two highly significant CMD resistance QTLs on chromosome 12 defining the *CMD2* locus, as well as additional putative QTLs on other chromosomes. Similar observations were made by Garcia-Oliveira et al. (2020) who found two closely linked loci on chromosome 12 and additional QTLs on chromosomes 9 and 10. Further dissection of the major QTL on chromosome 12 (*Manihot esculenta* v5.1 and 6.1 genome assembly) revealed the presence of two possible epistatic loci and/or multiple resistance alleles, which may account for the difference between moderate and strong disease resistance in the germplasm (Masumba et al., 2017; Nzuki et al., 2017; Garcia-Oliveira et al., 2020; Table 2). Gaps in the pseudochromosome 12 region containing the *CMD2* loci caused by highly repetitive DNA might explain the two separate loci (Kuon et al., 2019). Somo et al. (2020) identified QTLs associated with CMD severity across all chromosomes, except on chromosomes 4, 5, 6, 8, 11, 13, and 18. They validated the presence of the previously reported *CMD2* locus and detected another major QTL on chromosome 16. Nzuki et al. (2017) and Rabbi et al. (2020) confirmed the role of *CMD2* loci as the major gene for CMD resistance and reported two additional loci on chromosome 14 (Table 3).

**TABLE 3:** Summary of the GWAS studies of cassava.

Trait	Population/size	Markers used	Key findings	References
CMD	African breeding lines *n* = 6128	42 113 SNPs	A total of 198 SNPs mostly concentrated in a single region on Chr8, explaining 0.55 to 22% of PVE.	Wolfe et al., 2016
Dry matter content	Genetic Gain Collection *n* = 672	72 729 SNPs	Major locus at 24.1 Mbp of Chr1	Rabbi et al., 2017
Provitamin A			Major association regions at 24.1 and 30.5 Mbp on Chr1	
Root rot severity *Fusraium* (peel)	Cassava germplasm bank (EMBRAPA) *n* = 263	14 094 SNPs	Twenty-two (22) SNPs on Chr2, 5, 6, 8, 9, 10, 12, 17, 18, and ‘A fragments’ most significantly found on chromosome 10. The PVE ranged from 4.66 to 9.54%.	Brito et al., 2017
Root rot severity Fusarium (Pulp)			Eight (8) significant SNPs on Chr2,11.14,15,16. The most significant SNP was on Chr 11. The PVE range from 4.86 to 6.13%.	
Root rot severity *Phytophthora* (peel)			One (1) SNP on Chr13 explaining 7.99% of the phenotypic variation	
Root rot severity *Phytophthora* (peel)			One (1) SNP on Chr8 explaining 6.80% of the phenotypic variation.	
Root rot severity *Botryosphaeriaceae* (peel)			Three (3) SNPs on Chr5, 13, and genomic fragment “B” explain 8.90–10.09% of the phenotypic variation.	
Root rot severity *Botryosphaeriaceae* (pulp)			Three (3) SNPs on Chr9 and 12, explaining 5.47–7.04% of the phenotypic variation.	
Cassava green mite	Advanced breeding lines *n* = 845	61 307 SNPs	Twelve (12) significant loci on Chr8. The top significant explained 7% of the PVE.	Ezenwaka et al., 2018
Leaf pubescence			Seventeen (17) loci on Chr8. PVE ranged from 4 to 7%.	
Leaf retention			Five (5) loci on Chr8 each explaining 4% of the phenotypic variation.	
Stay green			One (1) significant SNP on Chr13 explaining 4% of the phenotypic variation.	
Carotenoid content	SC9 × white-root SC F_1_ (*n* = 98)	104 059 SNPs	Eighty-four (84) SNPs and 694 genes on all chromosomes except Chr5.	Luo et al., 2018
Storage root quality	Diversity Panel *n* = 158	19 850 SNPs	Four (4) association signals, including two SNPs on Chr5, and 15 for starch content and two SNPs on Chr14 and 16 for dry mass content.	Zhang et al., 2018
Yield components			Seven (7) association signals. Number of storage root (3, Chr4, Chr 5, Chr 9); storage root weight (1, Chr 2), dry mass weight (3, Chr 2).	
Morphological characteristics			Eleven (11) association signals. Stem diameter (6; Chr3, Chr6, Chr7, Chr14). First branch height (5; Chr2, Chr3, Chr9, Chr10).	
Leaf characteristics			Fourteen (14) association signals. Lobular length (4; Chr4, Chr5, Chr6, Chr18). Lobular width (3, Chr3, Chr5, Chr10). Petiole length (1, Chr1), Leaf aspect ratio (6, Chr1, Chr3, Chr6, Chr10, Chr14).	
CBSD foliar severity (3 months)	Diversity panel *n* = 1986	63 016 SNPs	Eighty-three (83) significant SNPs located on Chr11 the top SNPs explained 6% of the observed significant variation.	Kayondo et al., 2018
CBSD foliar severity (6 months)			The significant SNPs were located on Chr11, Chr4, and Chr12.	
All-*trans* β-carotene	GS training population *n* = 594	87 380 SNPs	Seven (7) significant SNPs with SNP effect ranging from 0.328 to 0.592.	Ikeogu et al., 2019
Lutein			Twelve (12) significant SNPs and 11 unique loci with SNP effects ranging from 0.025 to 0.034	
Total carotenoids content			Twenty (20) significant SNPs with SNP effect ranging from 0.435 to 0.749.	
Violaxanthin			One (1) significant SNP with an effect of 0.01	
13-*cis* beta-carotene			Twenty-three (23) significant SNPs with SNP effect ranging from 0.039 to 0.058.	
15-*cis* beta-carotene			Twenty-one (21) significant SNPs with SNP effect ranging from 0.008 to 0.011.	
9-*cis* beta-carotene			Eighteen (18) significant SNPs with SNP effects ranging from 0.032 to 0.045.	
CMD	Diverse breeding lines panels *n* = 432, *n* = 408, *n* = 402	84 434 SNPs	Genetic association detected on Chr1, 2, 3, 7,9,10, 12, 15, 15, 16, and 17. The presence of *CMD2* locus was validated. Another major CMD QTL was detected on Chr16.	Somo et al., 2020
CBSD root severity			Nine (9) SNPs on Chr2, 3, 8, 10 were significant.	
CBSDS| CMDS			One QTL on Chr4 and 2 on Chr12 significantly associated with both CBSDS/CMDS resistance.	
Waxy starch	Diversity panel *n* = 382	20,956 SNPs	10 association signals on Chr2	do Carmo et al., 2020
Cassava mosaic disease	Elite breeding clones *n* = 5130	101,521 SNPs	*CMD2* locus and other loci on Chr14 were captured. SNP effects ranged from −0.23 to 0.82	Rabbi et al., 2020
Cassava green mite			Four (4) regions associated with CMG on Chr1, 8, 12, 19. SNP effect size ranged from −0.18 to 0.10	
Apical pubescence			Significant association on Chr8, 9,11, 12, 16 with effect size from −0.19 to 0.08.	
Carotenoid content			Eight (8) significant associations on Chr1, 5, 8, 15, and 16 with SNP effect between −0.007 and 0.49. Major locus on Chr1 Around 24.1, 24.6 and 30.5 Mb.	
Dry matter content			Five (5) significant SNPs on Chr1, 6, 12, 15, 16. SNP effect between −1.32 and 0.85. The most significant SNP occurred on Chr1 around 24.64 Mb.	
Harvest index			Two (2) genomic regions associated with this trait on Chr2 and Chr12, with SNP effect of −0.04 and −0.03, respectively.	
Morphological traits			Eight (8) association signals detected. Outer cortex color 2 SNPs on Chr1 (3.05 Mbp) and Chr2 (6.56 Mbp). A single genomic region associated with periderm color on Chr3 (4.54 Mbp). 2 SNPs association signal for plant type on Chr1 (2.19 Mbp and 25.30 Mbp). Three Loci associated with stem color and Chr2 and Chrx 8 which contain the most significant at around 13.6 Mbp.	
Leaf morphology			Seven (7) association signals. Leaf petiole color single genomic region on Chr1 (23.45 Mbp); the same region was associated with mature leaf greenness. 3 association signals for apical leaf color (Chr2, 3, 8). For leaf shape two major loci on Chr 15 around 10.27 and 20.57 Mbp.	
Cyanogenic glycoside	Test population (*n* = 1246) Validation population (*n* = 636)	27,054 SNPS	Two significant peaks on chromosome 16 and 14, explaining 36 and 8% of the variance, respectively.	Ogbonna et al., 2020

*CBSD, Cassava Brown Streak Disease severity; CMD, Cassava Mosaic Disease.*

### Cassava Brown Streak Disease

Quantitative trait loci associated with CBSD root necrosis were identified on chromosomes 5, 11, 12, and 15 by Nzuki et al. (2017). The detected QTLs explained up to 10.18% of the PVE. Masumba et al. (2017) reported two consistent QTLs linked to resistance to CBSD-induced root necrosis on chromosomes 2 and 11, as well as a putative QTL on chromosome 18. Further additional putative QTLs were detected on other chromosomes (3, 4, 5, 6, 7, 10, 12, 15, and 16). In addition to QTLs found on chromosome 8 and 18, new putative QTL for CBSD root necrosis was identified on chromosome 14 (Garcia-Oliveira et al., 2020). Seven QTLs associated with CBSD foliar symptoms were found on chromosomes 4, 6, 15, 17, and 18. The most significant QTL explained 8.45% of the PVE (Nzuki et al., 2017). The study of Masumba et al. (2017) revealed several QTLs on all chromosomes linked with CBSD foliar symptoms, the most interesting of which was found on chromosome 2 and explained 4.6% of the PVE. Garcia-Oliveira et al. (2020) identified one major QTL on chromosome 18 that explained 12.87% of the phenotypic variation, but at a slightly different position. These same authors also reported a minor QTL on chromosome 11 (Table 2). The GWAS approach was used by Kayondo et al. (2018) to unravel the genetic architecture of CBSD. The polygenic control mechanism of resistance for CBSD and its instability across the environment was highlighted. Eighty-three (83) loci associated with foliar symptoms at 3 months after planting (MAP) were identified on chromosome 11. The top SNPs explained 6% of the phenotypic variance. Significant SNPs were identified on chromosomes 11, 4, and 12 for foliar severity score at 6 MAP. Recently, Somo et al. (2020) using a diverse panel of breeding lines, identified QTLs conferring resistance to CBSD on chromosomes 9 and 11 that accounted for 9 and 5% of PVE, respectively. Nine markers representing four loci on chromosomes 2, 3, 8, and 10 associated with resistance to CBSD for root necrosis were also reported by these authors (Table 2). Putative regions on chromosomes 11 and 15 were shown to be associated with both CBSD foliar and root necrosis symptoms (Nzuki et al., 2017), suggesting that CBSD root necrosis and CBSD foliar symptoms are most likely to be influenced to some extent by the same gene(s) or by closely linked genes at this locus. However, most of the QTLs reported have been associated with either root necrosis or foliar symptoms, supporting the notion that resistance to foliar and root symptoms of CBSD are largely under different genetic control (Masumba et al., 2017; Garcia-Oliveira et al., 2020). The detected QTLs were not consistent across studies. Different mapping populations were used in the case of experimental populations. There might be some variations in CBSD response. Furthermore, QTLs tend to be population specific in bi-parental populations. Likewise, different environmental conditions, population size (106–1986 samples), and the subjectivity of the scoring system used for data collection could have affected QTL detection and localization. The use of different versions of the cassava genome assembly makes it challenging to compare some of these results. Therefore, these QTLs should be mapped onto the most recent version of the cassava reference genome.

### Cassava Green Mite and Related Traits

Nzuki et al. (2017) detected on chromosomes 5 and 10 QTLs associated with cassava green mite (CGM) with maximum PVE of 10.56 and 10.08%, respectively. Recently, 95 SNP markers significantly associated with CGM resistance were reported by Ezenwaka et al. (2020). The significant markers concentrated in a single region of the left arm side on chromosome 12. The variance explained by the significant markers ranged from 18 to 31%. Garcia-Oliveira et al. (2020) detected five QTL for CGM resistance at 3 and 6 MAP, all with minor effect on chromosomes 5, 9, 13, and 18. While the QTLs on chromosome 9 was found in all four environment tested, the QTL on chromosome 8 co-localized with previously reported marker for CGM resistance (Table 2). The first GWAS to identify SNPs linked to CGM and CGM-related traits was performed by Ezenwaka et al. (2018) who found 35 significant SNP markers, including 12, 17, 5, and 1 associated with CGM, leaf pubescence, leaf retention, and stay green, respectively. All the significant markers were found on chromosome 8, except the SNP associated with stay green, which was identified on chromosome 13. Some of the significant SNP markers on chromosome 8 reported by these authors were also detected in the recent study of Rabbi et al. (2020) who identified other putative loci on chromosomes 1, 12, and 9. Association analysis of CGM-related traits, apical pubescence identified significant loci on five chromosomes, two of which co-located in the same regions underlying resistance to CGM on chromosomes 8 and 12 (Rabbi et al., 2020; Table 3).

### Cassava Bacterial Blight

Two QTLs associated with cassava bacterial blight (CBB) caused by *Xanthomonas axonopodis* pv. manihotis (*Xam*) were reported on linkage group (LG) 4 and LG8 that explained 12.6 and 10.9% of the field resistance to CBB, respectively (Sedano J. S. et al., 2017). Another study conducted by Sedano J. C. S. et al. (2017) found five strain-specific QTLs conferring resistance to *Xam* that explained 15.8 and 22.1% of the phenotypic variance. Three of the associated QTLs were found to be effective against *Xam*318 strain and explained 17.3 to 18.8% of the phenotypic variance, while two were detected for *Xam*681 and accounted for 15.8–22.1% of the phenotypic variance (Table 2).

### Cassava Root Rot

The complex nature of cassava root rot disease (CRR) was highlighted by Brito et al. (2017), who identified 38 significant SNPs associated with CRR. Of these, 8 and 22 were related to the severity of dry root rot in the pulp and peel, respectively, while the other eight were associated with soft root rot and black root rot (Table 3).

### Carotenoids and Storage Root Color

Three QTLs that control the content of carotenoids and four QTLs linked to the color of cassava roots pulp were identified by Morillo et al. (2013) (Table 2). Root yellowness resulting from carotenoid accumulation elucidated through GWAS has revealed major association regions that govern this trait around 24.1 and 30.5 Mbp of chromosome 1 (Rabbi et al., 2017). More recently, using a larger diversity panel, Rabbi et al. (2020) confirmed these previous findings. They found five new genomic regions associated with carotenoid content on chromosomes 5, 8, 15, and 16. Luo et al. (2018) identified 84 SNPs distributed in all chromosomes, except chromosome 5, associated with carotenoid traits. Ikeogu et al. (2019) identified 42 unique SNPs significantly associated with variation in total carotenoid content on chromosomes 1, 2, 4, 13, 14 and 15. Additional regions for variation in the total carotenoid content, as well as the individual carotenoids, were uncovered. Some regions associated with more than a single carotenoid were identified, suggesting the possibility of pleiotropic effects (Table 3). The level of phenotypic variability, the SNP frequencies and distributions, and the difference in sample size (98–5130 samples) between the panels used for GWAS might explain the differences in QTLs detected. The wider coverage of diversity could increase the detection of true novel associations, while small sample size could lead to some spurious associations.

### Cyanogenic Glucosides

Kizito et al. (2007) reported two QTLs (SSRY105, SSRY42) on two different linkage groups controlling cyanogenic glucosides. The two QTLs explained 7% and 20% of phenotypic variation, respectively. Also, both QTLs showed additive effects. Five QTLs associated with cyanogenic potential were identified across four linkage groups, including LG2, 5, 10, and 11 (Whankaew et al., 2011). The percentage of phenotypic variance explained from all detected QTLs ranged from 15.9 to 26.0% (Table 2). One (SSRY42) of the two QTLs reported by Kizito et al. (2007) was found in this latest study. More recently, Ogbonna et al. (2020) reported two genomic regions, in chromosomes 16 and 14, associated with hydrogen cyanide potential in cassava. The most significant marker was found on chromosome 16. Chromosomes 16 and 14 tagged SNPs explained 36 and 8% of phenotypic variance, respectively (Table 3). The significant region on chromosome 14 coincides with previously reported cyanide associated QTL reported by Kizito et al. (2007).

### Dry Matter Content

Six QTLs detected on four different LGs were reported to control DMC using a bi-parental mapping population. Individual QTL explained 14 to 40% of the variance. It was shown that additive, dominance, and overdominance effects play a role in the expression of this trait (Kizito et al., 2007; Table 2). Using genome-wide association mapping, DMC was found to be associated with a major locus occurring on a 24.1 Mbp region of chromosome 1 (Rabbi et al., 2017). This locus was confirmed by the recent study of Rabbi et al. (2020), who reported another major locus on chromosome 6 as well as three additional loci on chromosomes 12, 15, and 16 (Table 3).

### Starch and Starch Quality

The study of Thanyasiriwat et al. (2013) revealed the complex inheritance of starch pasting properties. Using average values of three environments, these authors reported 15 QTLs on LG1, 4, 6, 7, 8, 10 12, 13, 14, 16, and 18 affecting five starch pasting viscosities (peak viscosity, hot paste viscosity, cool paste viscosity, set back, and pasting temperature). The detected QTLs explained 10.0 to 48.4% of the phenotypic variance. Based on analysis of each environment, 48 QTLs significantly associated with seven starch pasting viscosities (peak viscosity, hot paste viscosity, break down, cool paste viscosity, setback, pasting time, and pasting temperature) were detected on all LGs except LG2, 4, and 7. The PVE ranged from 6.6 to 43.7. Thanyasiriwat et al. (2013) also reported two major QTLs on LG1 and LG6 for pasting temperature, which accounted for more than 70% of the phenotypic variation. Pootakham et al. (2014) identified a single co-localized QTL controlling pasting temperature and pasting time on LG7. The major QTL explaining 44.7 and 24.3% of the phenotypic variance. These authors reported additional QTL associated with starch pasting time on LG 10 that accounted for 22.5% of the phenotypic variance. A total of nine QTLs controlling fresh starch content was identified on seven linkage groups (LG4, 6, 7, 9, 11, 13, and 16). Among these, six QTLs were location-specific, and three QTLs were detected across three environments. The percentage of phenotypic variance explained by the QTLs ranged from 11.3 to 27.3% of the phenotypic variation (Sraphet et al., 2017; Table 2). Using GWAS, 10 SNP associated with waxy phenotypes were identified on chromosome 2 that co-located in genic regions that included five known genes and five genes of unknown function (do Carmo et al., 2020; Table 3).

### Agronomy and Physiology Traits

Eight QTLs associated with fresh root yield were identified on seven linkage groups (LG1, 2, 6, 9,12,13, and 16) from a bi-parental mapping population, of which two QTLs on linkage group 16 were found across two environments. These QTLs explained 12.9 to 40% of phenotypic variation (Sraphet et al., 2017; Table 2). Zhang et al. (2018) reported 36 loci related to 11 agronomic traits, including leaf characteristics, morphological characteristics, yield components, and root quality that were identified by GWAS analyses. They found seven SNPs associated with yield components that explained about 14.95% of the phenotypic variance on average. Morphological characteristics exhibited 11 association signals and explained 12.23 to 20.86% of the phenotypic variance. A total of 14 SNPs were identified from leaf characteristics, which explained 11.9 to 22.6% of the phenotypic variance. Genomic regions associated with harvest index were uncovered on chromosomes 2, 3, 4, 6, 8, 9, 12, 14, and 15. Two association signals for outer cortex color were found on chromosomes 1 (3.05 Mbp) and 2 (6.56 Mb). A single genomic region on chromosome 3 (4.54 Mbp) has been linked to periderm color. Two association signals were found for plant types on chromosome 1 (2.19 and 25.30 Mbp). Five loci associated with stem color variation were reported on chromosomes 2 and 8. The most significant loci were around 13.6 Mbp on chromosome 8. A single genomic region at around 23.45 Mbp on chromosome 1 was found concomitantly associated with leaf petiole color and mature leaf greenness. Variation in leaf color was found to be associated with three loci occurring on chromosomes 2, 3, and 8. At around 10.27 and 20.57 Mbp on chromosome 15, two loci associated were identified (Rabbi et al., 2020; Table 3). Seventy-one (71) markers significantly associated with leaf pubescence were identified by Ezenwaka et al. (2020) on chromosome 12. The variance explained by these significant markers ranged from 18 to 26%. The same study of Ezenwaka et al. (2020) reported 126 SNP markers associated with stay green on chromosome 12, and the variance explained by the detected QTLs ranged from 20 to 30% (Table 2).

Numerous favorable alleles, functional loci or regions linked to traits of interest have been identified through marker-trait associations and their phenotypic contribution identified, giving a glimpse to the genetics underlying phenotype variation. However, the designation of linkage groups/chromosomes are sometimes different, making it challenging to align QTL detected in different studies (Garcia-Oliveira et al., 2020). Synchronization of cassava QTL information and the development of a cassava QTL repository is needed. A cassava QTL database that contains QTL information systematically aligned to the cassava reference genome, as well as information about germplasm and genetic material used in the QTL studies, will be a useful resource for both cassava geneticists and breeders (Ni et al., 2009; Yonemaru et al., 2010; Said et al., 2015). A benefit envisioned from the identification of molecular markers tightly linked to traits of interest is their deployment in breeding as an indirect selection method to accelerate the rate of genetic gain.

## QTL Utilization in Cassava Breeding

Numerous QTL controlling a wide variety of traits have been identified to be utilized in marker-assisted selection (MAS) known as forward selection (Tables 2, 3). MAS is a technique of indirect selection of traits. Its implementation is still challenging in many breeding programs (Chukwu et al., 2019; Cobb et al., 2019). Although panels of molecular markers closely linked to key cassava traits have been identified, successful applied experiences of MAS in cassava breeding are limited (Table 4). Markers tightly associated with *CMD2* have been effectively used to identify genotypes bearing the *CMD2* gene in West African germplasm, introgression of *CMD2* genes into various cassava germplasm, and selection of parental lines for planned crosses. This has enabled breeders to focus on fewer genotypes at an early stage of the breeding program, saving the breeder time and labor cost (Blair et al., 2007; Okogbenin et al., 2012; do Carmo et al., 2015). MAS is appropriate for moderate to highly inherited traits that are controlled by a few major genes and not sufficiently predictive for quantitatively inherited traits controlled by many genes at different loci, each contribution to a small effect of the phenotypic expression (Singh et al., 2019). The time and investment required to develop reliable markers have also been a limiting factor for MAS implementation in cassava breeding programs in SSA. Likewise, the use of convenient phenotyping proxies do not always translate into meaningful selection targets for the breeding programs (Cobb et al., 2019). Future endeavors should prioritize cassava traits for marker development using a stage-gate system (SGS) to manage trait development. This will require the development of well-defined cassava product profiles, a set of targeted attributes the new variety should meet (Ragot et al., 2018). The establishment of a SGS will ensure accountability, transparency, and data-driven advancement decisions. This strategy is currently being implemented with the support of Excellence in Breeding (EiB)^1^. MAS should be considered only when proven more efficient than phenotypic selection to justify the expenses of development and deployment of the locus in a breeding program. Likewise, marker development for the traits retained should be feasible in a short to medium term to avoid waste of resources (Collard and Mackill, 2008). The International Institute of Tropical Agriculture (IITA) and partners have designed and converted molecular markers for several major gene traits, including provitamin A, CGM, DMC, to low-plex-high-throughput marker assay^2^. The reliability and utility of these markers are currently being evaluated in different genetic backgrounds and across different environments. A KASP assay has recently been employed to develop and validated diagnostic markers for HCN content (Ogbonna et al., 2020). The use of shared genotyping services could substantially reduce the genotyping costs, enabling the screening of a larger number of accessions at the seedling stage. Development of novel breeding strategies or models that not only capture additive effects, but also account for dominance and epistatic interactions, could contribute to the introgression of the QTLs into desired varieties. Likewise, the model should account for QTL-environment interactions for an effective MAS scheme (Singh et al., 2019). Recent genomic innovations have prompt for the search of new tools for population improvement.

**TABLE 4:** Key cassava traits, target regions, potential for GS and/or MAS and the current stage of trait development.

Traits	Region^*a*^	Priority^*b*^	Method of evaluation	Potential for MAS^*c*^	Stage 1^*d*^	Stage 2^*e*^	Stage 3^*f*^	Stage 4^*g*^
Cassava mosaic disease	All	High	Phenotyping/MAS/GS	High				X
Cassava brown streak	East Africa	High	Phenotyping/GS	Medium			X	
Cassava green mite	All	Medium	Phenotyping/MAS/GS	Medium				X
White flies	All	High	Phenotyping	Undetermined		X		
Pro-vitamin A	All	High	Phenotyping/MAS	High				
Low cyanogenic potential	East Africa	Medium	Phenotyping	High				
Root mealiness	East Africa and Central Africa	Medium	Phenotyping	High				
Dry matter content	All	High	Phenotyping/GS	Medium				X
Starch content	All	High	Phenotyping (low throughput)	Medium				X
Garri yield	West Africa	High	Phenotyping (low throughput)	Medium	X			
Fufu yield	West Africa	High	Phenotyping (low throughput)	Medium	X			
Plant type	All	High	Phenotyping/GS	Medium	X			
Harvest index	All	High	Phenotyping/GS	Low			X	
Root weight	All	High	Phenotyping/GS	Low			X	
Root number	All	High	Phenotyping/GS	Low			X	
Root size	All	High	Phenotyping/GS	Low			X	

*^*a*^Target regions; ^*b*^trait priority; ^*c*^potential for marker-assisted selection; ^*d*^Product scoping; ^*e*^Phenotyping and donor discovery; ^*f*^locus identification; ^*g*^Locus deployment; GS, Genomic selection; MAS, Marker-assisted selection.*

## Genomic Selection and It’s Potential in Cassava Breeding

Genomic selection (GS), also referred to as genomic prediction, can complement MAS; with MAS being used for highly heritable traits controlled by one or a few markers, and GS being used for more quantitative traits. GS relies on predicting the genomic estimated breeding values (GEBV) of an individual using a trait specific model built by simultaneously fitting information provided by thousands of molecular markers spread throughout the genome (Meuwissen et al., 2001). Fitting all markers simultaneously allows a substantial fraction of trait heritability missed by QTLs or association mapping to be captured. These are likely to be small effect alleles (Resende et al., 2012). The approach was first championed in dairy cattle (Jonas and de Koning, 2013). There are great expectations for the use of GS in cassava breeding. Since the first GS studies in cassava conducted by de Oliveira et al. (2012) and Ly et al. (2013), highlighting the potential of GS, several studies have been published (Wolfe et al., 2017; Ozimati et al., 2018; de Andrade et al., 2019; Yonis et al., 2020). GS has been utilized in cassava breeding to increase resistance against CMD and CBSD in cassava populations (Wolfe et al., 2016; Kayondo et al., 2018; Ozimati et al., 2018). Ozimati et al. (2018) highlighted the potential of GS as a pre-emptive breeding strategy. Torres et al. (2019) predicted good progress in selecting clones for traits such as fresh root yield (FRY), dry matter content (DMC), fresh shoot yield (FSY), harvest index (HI), dry yield (DY) using GS. Yonis et al. (2020) assessed genomic prediction for root size and shape based on root traits extracted from digital images. Greater predictive ability has been reported for DMC, CMD, and, to a lesser extent, HI compared to other traits (Wolfe et al., 2016; de Andrade et al., 2019). This success has been attributed to their high to moderate heritability, large-effect QTLs and low genotype × environment interaction (Torres et al., 2019; Yonis et al., 2020). GS is especially attractive for complex traits controlled by many QTLs with low heritability that are difficult or expensive to assess or are measured late in the breeding cycle (de Oliveira et al., 2012). It could drastically reduce the breeding cycle by choosing new parents based on GEBV rather than actual phenotypes and limiting the size and number of field experiments. However, refined strategies should be adopted for complex traits. Non-additive genetic variation prevails for low heritability cassava traits and should be accounted for. Therefore, models that capture non-additive effects should be applied (Wolfe et al., 2016). GS, like any other approaches, is facing various challenges. The GS models predict poorly across populations, and consequently, the strategy requires continuous re-calibration with every breeding cycle (Wolfe et al., 2017). Allele frequency changes, introgression of new alleles, SNP-QTL linkage disequilibrium association, lack of relatedness between germplasm and population structure, as well as genotype-by-environment interaction effects, compromise the efficiency of GS. To overcome some of these limitations, dual-purpose population development and variety development pipelines have been applied to ensure training data are closely related to the new clones and for continuously updating the training model (Santantonio et al., 2020). GS could be more robust by integrating biological knowledge. The inclusion of QTL markers associated with the trait of interest could increase the robustness of genetic evaluation (Ozimati et al., 2018; Lan et al., 2020). Other variables affecting the precision of the prediction model include the size of the training population, the number of markers used in the model, the trait genetic architecture, and heritability (de Oliveira et al., 2012; Ly et al., 2013; Wolfe et al., 2017; Somo et al., 2020). Poor predictions have been reported across breeding programs limiting the prospect of sharing data from different locations, breeding programs, and countries (Wolfe et al., 2017; Somo et al., 2020). An international project funded by UK Foreign, Commonwealth and Development Office (FCDO) and the Bill and Melinda Gates Foundation (Gates Foundation) named the Next Generation Cassava Breeding Project^3^ is currently using this approach in four African research institutes, including the National Crop Resources Research Institute (NaCRRI) in Uganda, the National Root Crops Research Institute (NRCRI) in Nigeria, the Tanzania Agriculture Research Institute (TARI) in Tanzania, and IITA in Nigeria. A multi-trait genomic selection strategy is being applied using program-based selection indices to efficiently improve quantitative traits simultaneously. The selection is based on yield, DMC, virus resistance to CMD and CBSD, and good plant architecture. Outstanding clones are currently advanced toward the variety release pipeline. The adoption of GS has guided the mating design, enabling rapid pyramiding of favorable allele combinations and development of progeny with the improved allelic combinations (de Oliveira et al., 2012). Moreover, GS is being used to increase genetic gain by decreasing the breeding cycle time, increasing selection accuracy, and increasing selection intensity in early generations. In order to identify a more effective breeding strategy and further improve breeding efficiency, computational simulations are currently being performed with the support of EIB^4^ to: (1) compare different selection methods; (2) define optimal crossing strategies; and (3) determine appropriate recycling times. The data intensive nature of GS and widespread use of this approach across more breeding programs requires development of shared computational infrastructures and analysis pipelines.

## Development of Computational Tools for a Greater Impact

Breeding modernization will necessitate the development of robust statistical tools and innovative analytical pipelines and platforms to accommodate the enormous quantity of data, which is being generated. Due to its clonal propagation method, there is a narrow timeline between harvesting and the next cassava planting season. This necessitates quick decision making and effective data management. Information systems are required to track samples, store genomic and phenotypic information, merge data, and conduct analysis to guide decision making (Santantonio et al., 2020). It is from this perspective that an open access repository, such as Cassavabase^5^, has been developed to centralize information access to cassava research data and support cassava breeding programs (Fernandez-Pozo et al., 2015). Cassavabase contains phenotypic, pedigree, and genomics data generated over 30 years by cassava programs in Africa, Asia, and South America. Cassavabase also encompasses computational tools to facilitate analyses.

## Genome Editing Advances and Prospects

Genome-editing is becoming a popular molecular tool for functional genomics, as well as crop improvement. Clustered regularly interspaced palindromic repeats (CRISPR/CRISPR-associated protein 9 (Cas9)-mediated genome-editing has rapidly become the most popular genome engineering approach due to its simplicity, efficiency, specificity, multiplexing and ease to adapt. Most of the CRISPR/Cas9-based genome-editing is reported for seed crops; however, recently, it is also established for clonally propagated crops such as potato, banana, and cassava (Butler et al., 2016; Odipio et al., 2017; Kaur et al., 2018; Naim et al., 2018; Tripathi et al., 2019; Ntui et al., 2020). The accessibility of the genetic transformation system and reference genomes for cassava made it possible to realize the potential of CRISPR-based genome-editing for basic and applied research to improve economically important cassava traits. The CRISPR/Cas9-based genome-editing system was demonstrated by knocking out the *Phytoene desaturase* (*MePDS*) gene in two cultivars of cassava, TMS60444 and TME204 (Odipio et al., 2017). Genome-editing was further applied for developing a cassava variety with resistance against two species of Ipomovirus, *cassava brown streak virus* (CBSV) and *Ugandan cassava brown streak virus* (UCBSV), causing CBSD (Gomez et al., 2019). The targeted mutations in the translation initiation factor 4E (eIF4E) isoforms nCBP-1 and nCBP-2 in the edited cassava variety showed a reduction in disease severity and virus accumulation in the storage tuberous roots upon glasshouse challenge of edited cassava lines with CBSV. Even though the mutations in the nCBP-1 and nCBP-2 genes conferred enhanced resistance to CBSD, complete resistance was not obtained. This suggests that total resistance to CBSD can be developed by stacking the genome-editing approach of disrupting eIF4E isoforms with other resistance strategies such as RNAi (Gomez et al., 2019). Later, researchers have attempted to apply this technology to develop resistance against a geminivirus, *African cassava mosaic virus* (ACMV) (Mehta et al., 2019). However, the edited cassava plants did not show significant resistance against ACMV in greenhouse inoculation experiments. It might be due to the evolution of editing-resistant geminiviruses in genome-edited cassava. CRISPR/Cas9 based genome-editing can be coupled to genetic improvements in cassava for traits such as starch improvement and early flowering. Cassava roots normally produce large quantities of starch, having high amylose levels, a crystallizable component that is more soluble in water. However, the starch with low amylose levels, known as “waxy starch,” is preferred for food processing and other industrial uses. Bull et al. (2018) reported the application of CRISPR/Cas9 for manipulating starch biosynthesis and improving the starch quality in the storage. They generated edited cassava plants with mutations in two genes: *protein targeting to starch* (PTST1) or *granule bound starch synthase* (GBSS) involved in amylose biosynthesis, leading to reduction or elimination of amylose content. This, in turn, can improve the quality of starch cassava roots for commercial use. The authors also demonstrated accelerated breeding by transferring the *Arabidopsis* FLOWERING LOCUS T gene in the genome-edited events of cassava for early flowering. Genome-editing can multiplex the traits, and researchers can develop cassava varieties with the waxy starch and early flowering. Despite still being in its infancy, genome-editing offers promising prospects for cassava improvement and could shorten the breeding process. Novel plant varieties could be directly used for crop production or as pre-breeding materials (Xu et al., 2019). Technological developments like this should be followed by their adoption to increase productivity.

## Current Status of Technology Applications in Sub-Saharan Africa

Effective implementation of technological development is key to enhancing the productivity and profitability of cassava on the continent. Genetic studies conducted by national institutes or academia in sub-Saharan Africa have been mainly focused on germplasm characterization, genetic diversity assessment, varietal identification, linkage mapping, and classical QTL mapping (Fregene et al., 1997; Kawuki et al., 2009; Rabbi et al., 2015; Adjebeng-Danquah et al., 2020). Recently, collaborative projects involving international institutions, NARS, along with substantial funding from the donor communities, have contributed to the increased number of genome-wide association studies. Several gene(s)/QTLs underlying key cassava traits have been identified and trait-linked markers, a pre-requisite for MAS have been developed (Ogbonna et al., 2020; Rabbi et al., 2020). The effective implementation of MAS hampered by economic obstacles is currently being addressed with the support of the EIB platform^2^, who seeks to mainstream the use of genotyping data. The platform through subsidies from the Gates Foundation offers small breeding programs access to high quality genotyping services, including low-density (Kompetitive allele specific PCR (KASP)) and mid-density (DArTAg) genotyping to foster the progressive integration of MAS into NARS crop breeding programs. The EIB low-density genotyping service is being used by IITA and NARS partners in Uganda, Tanzania, and Nigeria for quality control, identification of cassava accessions with the desired alleles, and validation of trait-markers. Genomic selection-based pilot projects are ongoing in Nigeria, Uganda, and Tanzania and the appealing perspectives offered by this approach, including shortening of the breeding cycle and speed-up of variety development have been highlighted (Wolfe et al., 2017; Kayondo et al., 2018; Somo et al., 2020). The mid-density genotyping, DArTAg, a target genotyping provided by Diversity Arrays is being used as an alternative to GBS for genomic selection applications. Although traditional methodologies are still widely used for trait phenotyping, integration of high throughput phenotyping is on course. NIRS spectroscopy is being explored by few NARS programs to predict some key cassava traits (i.e., carotenoids and DMC) and promising results have been reported (Ikeogu et al., 2017, 2019). NIRS evaluation and optimization for other traits, including gari, fufu, starch, and root mealiness is ongoing. In collaboration with IDS GeoRadar^6^, IITA is testing a prototype commercial GPR for routine cassava root phenotyping. The unmanned aerial vehicle (UAV) is currently used at IITA by the Cassava Source-Sink Project to quantify aboveground plant growth (Sonnewald et al., 2020). Image recognition has been evaluated for high accuracy disease detection and mobile, as well as web-based tools developed for cassava disease scoring and monitoring^7^. An application programming interface has been implemented within Cassavabase in order to process cassava root images. Breeders can upload cassava root necrosis sectional image captured during harvest and the result will be returned back to Cassavabase. IITA and NARS partners have digitized all processes; data are uploaded, processed, and accessed within Cassavabase to minimize human errors. CRISPR/Cas9-based genome-editing is mainly driven by IITA, in partnership with a handful of national research organizations. The difficulty in acquiring laboratory supplies, as well as the need for constant and sufficient level of funding, constitute a bottleneck for the implementation of gene-editing technology by NARS. Likewise, the legal framework and appropriate regulatory structures needed to guide the use of this technology are still lacking in several African countries, hampering the movement of plants from laboratory to the field (Tripathi et al., 2020). Furthermore, biosafety considerations remain a public concern. Winning consumer’s acceptance and trust is key to the effective implementation of this new breeding technique and to harness its full potential. Few cassava breeding programs have benefited and/or explored these recent technological developments. For greater impact, the knowledge and technologies outlined here should be disseminated and transferred to other cassava breeding programs on the continent.

## Technology Transfer and Capacity Development for More Sustainable Cassava Production

Sustained research and innovation capacity is imperative for agricultural transformation in Africa (Ojijo et al., 2016). Most African national agricultural research systems (NARS) do not have well-funded cassava breeding programs with sufficient technical critical mass. Many programs routinely evaluate and multiply clones imported from larger breeding programs (i.e., from IITA or CIAT). Although most NARS would maintain certain capacity to develop and release varieties adapted to local agro-ecologies and local preferences, their breeding capacities need to be strengthened and modernized to be effective. Many of the technological innovations are new to NARS and access to equipment, reagents, and skilled personnel is challenging (Tester and Langridge, 2010). It is with this in mind that an initiative such as the Cassava Community of Practice and Partnership (CoPP) has been established through the NextGen Cassava Project to serve as a platform to disseminate and facilitate the transfer of proven tools, methods, technologies, and products. The CoPP provides the initial technical backstopping, fosters collaboration, facilitates connectivity, and creates opportunities for peer-to-peer learning between members. This will enable the establishment of best practices and procedures and implementation of recent technological innovations by NARS, increase breeding efficiencies, and create a broader and deeper impact. Successful integration and application of innovative technologies necessitate technical expertise. Scaling up the research capabilities of NARS, through training, will hone their skills and knowledge (Bull et al., 2011). The CoPP concept is not new. A successful example is the sweet potato breeding Community of Practice, which has strengthened national capacities leading to the development and released of several user-preferred varieties with impact on the quality of life of small farmers (SASHA, 2019). The cassava CoPP will need to evolve into a cohesive network with clear accountabilities and expectations. NARS and CGIAR will need to work together in a coordinated breeding network for economies of scale. Government involvement will be needed to sustain the gain achieved by the breeders.

## Conclusion and Perspectives

It takes several years to develop improved cassava varieties. Genomic resources have facilitated the evaluation of local and regional genetic diversity and profiling of breeding materials. New phenotyping approaches are substituting traditional trait evaluation and have been used for marker-trait associations, enabling identification of numerous QTLs for key traits. Although notable signs of progress have been achieved, especially for less complex traits, genetic architecture is still not fully understood. For quantitatively inherited traits, phenotypic plasticity and the difficult phenotypic evaluation complicates matters further. There is a need to scale up multi-environment evaluation. The development of advanced high throughput and accurate cassava phenotyping approaches is imperative. Translating those results obtained into practical breeding methodologies and coherent biological knowledge is needed. QTL results should be exploited, and validated trait-markers developed. Priorities will have to be set to ensure return on investment. Therefore, establishment of a formal advancement system with well-defined metrics is needed. Genetic gain should be routinely monitored. Strategies need to be constantly revised as priorities evolve, and new challenges emerge. MAS and GS are complementary breeding approaches that should be used in tandem. More attention should be given to quality traits, which have received less attention; whereas quality traits influence varietal adoption and product utilization. Key quality traits need to be defined and translated into biochemical parameters. Advanced technologies, such as genome-editing, could play a prominent role in cassava improvement. However, further investigation will be required to ensure maximum benefits. It will be important that the new technologies and tools developed are used by NARS. Therefore, regional networks, shared expertise, and service will be of prime importance. Last, but not the least, the support and involvement of national and regional governments is crucial for sustainable cassava production on the continent.

## Author Contributions

EN conceived this review and drafted the manuscript. CE conceived the idea and edited the manuscript. LT drafted a section of the manuscript. MF, IR, EH, KSI, PK, and CE provided critical edits. All authors contributed to the article and approved the submitted version.

## Conflict of Interest

The authors declare that the research was conducted in the absence of any commercial or financial relationships that could be construed as a potential conflict of interest.

## Footnotes

1 https://excellenceinbreeding.org/blog/applying-stage-gates-better-manage-public-breeding-programs

2 https://excellenceinbreeding.org/module3/kasp

3 NextGen cassava, https://www.nextgencassava.org

4 https://excellenceinbreeding.org/module2

5 https://www.cassavabase.org/

6 https://idsgeoradar.com/

7 http://www.mcrops.org/
